# Impact of comorbidity on the short- and medium-term risk of revision in total hip and knee arthroplasty

**DOI:** 10.1186/s12891-020-03455-3

**Published:** 2020-07-09

**Authors:** Jorge Arias-de la Torre, Kayla Smith, Alexandru Dregan, Jose M. Valderas, Jonathan P. Evans, Daniel Prieto-Alhambra, Luis Lozano, Antonio J. Molina, Vicente Martín, Laia Domingo, Laura Muñoz, Mireia Espallargues

**Affiliations:** 1 Agency for Heath Quality and Assessment of Catalonia (AQuAS), Carrer de Roc Boronat, 81, 08005 Barcelona, Spain; 2 King’s College London, Institute of Psychiatry, Psychology and Neuroscience (IoPPN), London, UK; 3 CIBER Epidemiology and Public Health (CIBERESP), Madrid, Spain; 4 Institute of Biomedicine (IBIOMED, University of Leon, León, Spain; 5 Health Services Research on Chronic Patients Network (REDISSEC), Madrid, Spain; 6 Health Services and Policy Research Group, University of Exeter Medical School, Exeter, UK; 7 Royal Devon and Exeter NHS Foundation Trust, Exeter, UK; 8 Oxford NIHR Musculoskeletal Biomedical Research Unit, Nuffield Department of Orthopaedics, Rheumatology and Musculoskeletal Sciences, University of Oxford, Oxford, UK; 9 Hospital Clinic de Barcelona, Barcelona, Spain; 10 Department of Epidemiology and Evaluation, IMIM (Hospital del Mar Medical Research Institute), Barcelona, Spain

**Keywords:** Total knee arthroplasty, Total hip arthroplasty, Comorbidity, Arthroplasty revision, Register studies

## Abstract

**Background:**

The impact of comorbidity on the risk of revision in patients undergoing Total Knee arthroplasty (TKA) and Total Hip Arthroplasty (THA) is not currently well known. The aim of this study was to analyze the impact of comorbidity on the risk of revision in TKA and THA.

**Methods:**

Patients recorded in the Catalan Arthroplasty Register (RACat) between 01/01/2005 and 31/12/2016 undergoing TKA (*n* = 49,701) and THA (*n* = 17,923) caused by osteoarthritis were included. As main explanatory factors, comorbidity burden was assessed by the Elixhauser index, categorized, and specific comorbidities from the index were taken into account. Descriptive analyses for comorbidity burden and specific conditions were done. Additionally, incidence at 1 and 5 years’ follow-up was calculated, and adjusted Competing Risks models were fitted.

**Results:**

A higher incidence of revision was observed when the number of comorbidities was high, both at 1 and 5 years for THA, but only at 1 year for TKA. Of the specific conditions, only obesity was related to the incidence of revision at 1 year in both joints, and at 5 years in TKA. The risk of revision was related to deficiency anemia and liver diseases in TKA, while in THA, it was related to peripheral vascular disorders, metastatic cancer and psychoses.

**Conclusions:**

Different conditions, depending on the joint, might be related to higher revision rates. This information could be relevant for clinical decision-making, patient-specific information and improving the results of both TKA and THA.

## Background

Total knee and hip arthroplasties (TKA and THA) are safe, successful, and cost-effective treatments for late-stage osteoarthritis in the knee and hip that offer pain relief, better function and improved quality of life to the patient [1–5]. These procedures have increased worldwide in the last few decades [2, 6] and are expected to rise due to the increase in life expectancy and prevalence of osteoarthritis [7, 8]. Previous research shows that certain physical and mental comorbidities that are particularly prevalent in the elderly population could have a greater risk of complications (medical, surgical, and wound) following TKA and THA [3, 9].

TKA and THA are common considerations for effective procedures for patients with advanced osteoarthritis [7, 10]. Although using comorbidities to predict clinical outcomes has increased substantially in the past 20 years for patients undergoing TKA and THA [2, 6, 11, 12], their relationship to the risk of revision is unclear. Moreover, most joint arthroplasty patients have a high prevalence of comorbidities, which can affect surgical outcomes, and previous studies have identified risk factors associated with lower implant survival [13]. Revision surgery is a more demanding procedure than a primary total joint arthroplasty (TJA) and thus requires more extensive resources [14]. Furthermore, late revisions are becoming more common for patients, given the finite life of an implant, coupled with the increased use of THA in younger patients [15].

Several indices are currently available to assess the risk of different outcomes, such as prosthesis revision, mortality and Patient Reported Outcome Measures (PROMs), based on the presence of comorbidities for patients undergoing TKA and THA. Despite this, there is still no consensus on an optimal approach for risk adjustment [16], but the Charlson Comorbidity Index (CCI) and the Elixhauser Comorbidity Index are the two most commonly used [17]. The Elixhauser index, introduced in 1998 [18], is a composite measurement to assess the impact of comorbidity on various surgical procedures [19]. The original version of this index included 30 burdensome conditions [7, 17, 18], like hypertension, obesity, weight loss, and psychiatric disorders, [8, 15, 20, 21] that have proven to be particularly relevant in TKA and THA, due to their relationship with prosthesis revision. Given that the aforementioned comorbidities are excluded from the CCI [16], growing evidence suggests that the Elixhauser method is preferable for risk-adjustment in orthopedic surgery [19].

Previous research has identified patient factors like age, sex, obesity, and hospital characteristics that may increase the revision risk for TKA [22]. An in-depth understanding of how comorbidity might influence the incidence of revision should take these risk factors into account to ensure the most accurate estimates possible. This information could be useful in guiding clinical decision-making, and consequently might reduce the burden of revision surgery. Therefore, the aims of this study were: 1) to examine patient characteristics and comorbidity distribution in those undergoing TKA and THA; and 2) to analyze the impact of the burden of comorbidity, and that of the specific comorbidities in the Elixhauser index, on the risk of revision at 1 and 5 years in patients undergoing TKA and THA.

## Methods

### Study design and population

A retrospective observational study based on data from the Catalan Arthroplasty Register (RACat) and the Minimum Basic Dataset at Hospital Discharge (MBD-HD) was carried out. The RACat is a population-based hip and knee arthroplasty register that has collected information since January 2005 on those types of surgical procedures and their related aspects not accounted for in the MBD-HD (e.g. laterality of the arthroplasty, fixation type or the bearing surface in THA). The RACat includes 52 out of 55 public hospitals in Catalonia that perform knee and hip arthroplasty surgery, with an overall completeness of about 90% for primary arthroplasties and about 70% for revision procedures [23].

The MBD-HD is a mandatory, payment-related administrative database that provides information about different aspects of the patient and surgical procedures, like the patient’s main diagnosis and up to 10 secondary diagnoses based on ICD-9-CM codes, as well as patient characteristics and the causes of primary and revision interventions. This dataset is linked to the RACat dataset through a patient identification number and other variables like the hospital admission, surgery and hospital discharge dates. Additionally, given that the MBD-HD is a payment-related dataset and all procedures performed in public hospitals should be reported, it was used as a standard to calculate the completeness of the RACat.

### Study variables

For the present study, the first revision of the primary TKA and THA was considered as the main outcome. A revision arthroplasty was defined by the RACat as any procedure involving removal, exchange or addition of any implant component. Patient follow-up started 1 January 2005 and ended 1 January 2017.

Both comorbidity burden and the specific comorbidities at the time of the primary intervention were considered as main explanatory variables. The specific conditions included in the Elixhauser index were evaluated. The Elixhauser index is a composite measurement of comorbidity based on the Clinical Modification of the ninth version the International Classification of Diseases (ICD-9-CM) codes [7, 18]. The index is made up of 30 categories, each corresponding to a group of specific diagnoses, including the following: hypertension without complications, diabetes without complications, obesity, depression, chronic pulmonary disease, hypothyroidism, deficiency anemia, renal failure, hypertension with complications, valvular disease, rheumatoid arthritis /collagen vascular disease, other neurological disorders, liver disease, congestive heart failure, peripheral vascular disorders, solid tumor, diabetes with complications, coagulopathy, blood loss anemia, fluid and electrolyte disorders, alcohol abuse, psychoses, paralysis, pulmonary circulation disorders, lymphoma, peptic ulcer disease (excluding bleeding), drug abuse, metastatic cancer, weight loss and AIDS. The comorbidity burden was considered as the number of specific comorbidities that each individual presented from the index at the time of the primary intervention. Based on the distribution of the number of comorbidities in the studied population, with a strong positive asymmetry, comorbidity burden was considered a categorical variable using tertiles as cut-off values. The following levels were considered: 0 comorbidities, 1 comorbidity and 2 or more comorbidities. Additionally, each of the specific comorbidities composing the index was considered separately.

The following covariates were also taken into account: sex (male and female); age in years (continuous); prosthesis fixation (cemented, when the stem and cup in THA were cemented and the tibial and femoral components in TKA; uncemented when none of the prosthesis components were cemented; hybrid when the stem in THA was cemented but not the cup, and the tibial but not the femoral component in TKA; and inverse hybrid when the cup but not the stem was cemented in THA and the femoral but not the tibial component was cemented in TKA); year of intervention (categorized as 2005–2008, 2009–2011, 2012–2014 and 2015–2017) and type of hospital, according to the range of services offered in the hospital itself, irrespective of the patient’s territorial assignment (high technology, reference, regional and other type or not specified) [24].

### Statistical analysis

Descriptive analyses of patient characteristics were done as well as a description of the absolute and relative frequencies of patients in the different levels of comorbidity burden. A description of these frequencies in the specific comorbidities included in the Elixhauser index was also given.

Absolute and relative frequencies of revision in patients that presented comorbidity (for burden and for the specific comorbidities) throughout the entire study period (2005 to 2016) were obtained, and the incidence of revision at 1 and 5 years was calculated, considering patient death as the competing event of primary arthroplasty revision. Taking this into account, the incidence of revision up to the 1st of January 2017 was estimated by calculating t S (t-1) * h’ (t), where S (t-1) was the Kaplan-Meier estimate of the overall survival function and h’ (t) was the cause-specific hazard at time t. To assess the relationship between comorbidity and risk of revision, adjusted Fine and Gray Competing Risk regression models were fitted. From these models, Subhazard Ratios (SHR) and their respective 95% Confidence Intervals (95% CI) were obtained. All models were fitted separately for short-term (1 year) and medium-term (5 years) follow-up, and were adjusted for all patient characteristics. The absence of interactions between explanatory variables and multi-collinearity was verified. Absence of interactions was assessed using a likelihood ratio test to compare the models including all covariates and all second-grade possible interactions with a model including all covariates without interactions. As the results were non-significant, the absence of significant interactions was assumed. Absence of multi-collinearity was assessed looking at the correlation matrix of the estimates from models and using variance inflation factor tests (VIFs). All analyses were stratified by joint, TKA and THA, and were carried out independently for comorbidity burden and for each comorbidity in the Elixhauser index. The statistical significance level was fixed at α = 0.05 and all analyses were done using the statistical software Stata v.14.

## Results

### Patient characteristics and comorbidity distribution in the study population

**Fig. 1:**
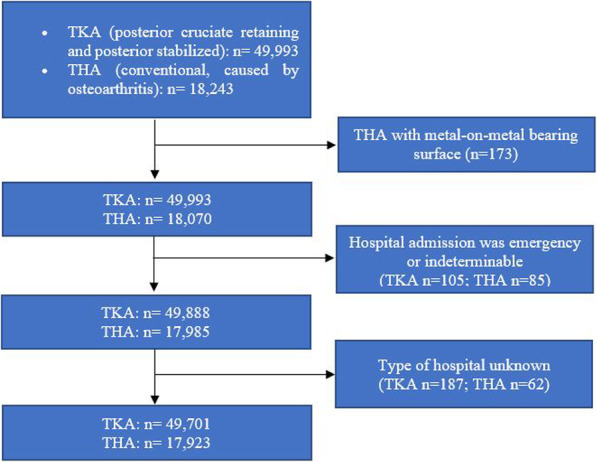
Selection of the study sample

All patients undergoing TKA (posterior cruciate retaining and posterior stabilized) and THA (conventional, excluding those with metal-on-metal bearing surface *n* = 173, 0.9%) caused by osteoarthritis (OA) and recorded in the RACat from 01/01/2005 to 31/12/2016 were included in the study population (TKA *n* = 49,993; THA *n* = 18,070) (Fig. 1). Patients with emergency indeterminable hospital admission (TKA *n* = 105, < 0.2%; THA *n* = 85, 0.5%) or for whom the type of hospital in which they were operated on was unknown (TKA *n* = 187, 0.4%; THA *n* = 62, 0.3%) were excluded from the study, thus yielding a total of 49,701 TKA and 17,923 THA procedures included for analysis. The total number of deaths was 3242 (6.5%) for TKA, and 1372 (7.7%) for THA.

Table 1 shows the general characteristics of the study population separately for TKA (*n* = 49,701) and for THA (*n* = 17,923). The most frequent characteristics for TKA and THA, respectively, are: female (TKA 70.9% and THA 50.6%), between 65 and 74 years old (TKA 43.5% and THA 34.6%), operated on between 2011 and 2013 (TKA 29.1% and THA 33.5%), having a prosthesis with cemented fixation type in TKA (76.4%) and uncemented in THA (68.0%) and operated on in a reference hospital (TKA 42.9% and THA 45.1%). Table 2 shows the distribution of comorbidity burden and the specific comorbidities in the Elixhauser index and their frequencies of revision during the study period (2005–2016). In both joints, the most frequent specific comorbidities were hypertension without complications (TKA 54.3% THA 44.5%) and diabetes without complications (TKA 15.9% THA 12.4%). The specific comorbidities with the highest revision rates were liver diseases in TKA (5.1%) and peripheral vascular disorders in THA (4.2%). In TKA depression was the third most common comorbidity (9.5%) and the least common comorbidity was AIDS, present in only 4 patients (< 0.1%). In THA, the third most common comorbidity was hypothyroidism (7.9%) and the least common, peptic ulcer disease excluding bleeding, present in only 8 patients (< 0.1%).

**Table 1:** General characteristics of the study population by comorbidity burden (2005–2016)

	TKA (***n*** = 49,701)		THA (***n*** = 17,923)	
	n	%	n	%
**Sex**				
Men	14,455	29.1	8854	49.4
Women	35,246	70.9	9069	50.6
**Year of the intervention**				
2005/2007	6883	13.9	2060	11.5
2008/2010	14,309	28.8	4939	27.6
2011/2013	14,457	29.1	6015	33.5
2014/2016	14,052	28.3	4909	27.4
**Fixation type**				
Cemented	37,950	76.4	1596	8.9
Uncemented	353	0.7	12,186	68.0
Hybrid	7274	14.6	3895	21.7
Inverse hybrid	442	0.9	197	1.1
Not specified	3682	7.4	49	0.3
**Type of hospital**				
High technology hospital	19,066	38.4	6416	35.8
Reference hospital	21,337	42.9	8089	45.1
Regional hospital	9298	18.7	3418	19.1
**Median age in years (IQR)**	73.0 (10.3)		71.4 (14.2)	
**Median follow-up in years (IQR)**	4.7 (5.1)		4.6 (4.6)	
**Median number of comorbidities (IQR)**^a^	1 (2)		1 (2)	

*TKA* Total Knee Arthroplasty, *THA* Total Hip Arthroplasty

n: number of patients

%: percentage of patients

*IQR* Inter-quartile range

^a^min and max 0 and 7 respectively both for TKA and THA

**Table 2:** Distribution of burden and specific comorbidities in the population from the Elixhauser index (2005–2016)

	TKA (***n*** = 49,701)				THA (***n*** = 17,923)			
	Primary		Revision		Primary		Revision	
	n	%	n	%	n	%	n	%
**Burden of comorbidity**								
0 comorbidities	14,548	29.3	528	3.6	6862	38.3	175	2.5
1 comorbidity	17,479	35.2	585	3.3	6079	33.9	159	2.6
> =2 comorbidities	17,674	35.6	531	3.0	4982	27.8	130	2.6
**Uncomplicated hypertension**	26,968	54.3	837	3.1	7972	44.5	209	2.6
**Uncomplicated diabetes**	7903	15.9	221	2.8	2223	12.4	63	2.8
**Obesity**	5383	10.8	180	3.3	1235	6.9	46	3.7
**Depression**	4735	9.5	159	3.4	1160	6.5	33	2.8
**Chronic pulmonary disease**	4100	8.3	117	2.9	1420	7.9	33	2.3
**Hypothyroidism**	2928	5.9	93	3.2	746	4.2	16	2.1
**Deficiency anemia**	1162	2.3	34	2.9	471	2.7	12	2.5
**Renal failure**	964	1.9	18	1.9	391	2.2	7	1.8
**Hypertension with complications**	949	1.9	18	1.9	305	1.7	2	0.6
**Valvular disease**	886	1.8	22	2.5	300	1.7	2	0.7
**Rheumatoid arthritis /collagen vascular disease**	680	1.4	19	2.8	288	1.6	7	2.4
**Other neurological disorders**	724	1.5	22	3.0	233	1.3	5	2.1
**Liver disease**	673	1.4	34	5.1	284	1.6	8	2.8
**Congestive heart failure**	489	1.0	8	1.6	158	0.9	2	1.2
**Peripheral vascular disorders**	368	0.7	11	3.0	166	0.9	7	4.2
**Solid tumor**	226	0.5	3	1.3	100	0.6	2	2.0
**Diabetes with complications**	227	0.5	13	5.7	64	0.4	NR	NR
**Coagulopathy**	221	0.4	3	1.4	77	0.4	NR	NR
**Blood loss anemia**	215	0.4	13	6.1	67	0.4	NR	NR
**Fluid and electrolyte disorders**	214	0.4	5	2.3	126	0.7	2	1.6
**Alcohol abuse**	194	0.4	6	3.1	132	0.7	3	2.2
**Psychosis**	172	0.4	3	1.7	77	0.4	5	6.5
**Paralysis**	88	0.2	7	8.0	39	0.2	2	5.1
**Pulmonary circulation disorders**	75	0.2	2	2.7	20	0.1	NR	NR
**Lymphoma**	38	0.1	1	2.6	16	0.1	1	5.9
**Peptic ulcer disease (excluding bleeding)**	31	0.1	2	6.5	8	< 0.1	NR	NR
**Drug abuse**	12	< 0.1	NR	NR	28	0.2	NR	NR
**Metastatic cancer**	10	< 0.1	NR	NR	10	0.1	1	10.0
**Weight loss**	10	< 0.1	1	10.0	9	0.1	NR	NR
**AIDS**	4	< 0.1	NR	NR	16	0.1	1	5.9

*THA* Total Hip Arthroplasty, *TKA* Total Knee Arthroplasty

n: total number of patients in primary or revision THA or TKA during the study period (2005–2016)

%: percentage of patients

### Influence of comorbidity on the risk of revision in TKA

**Fig. 2:**
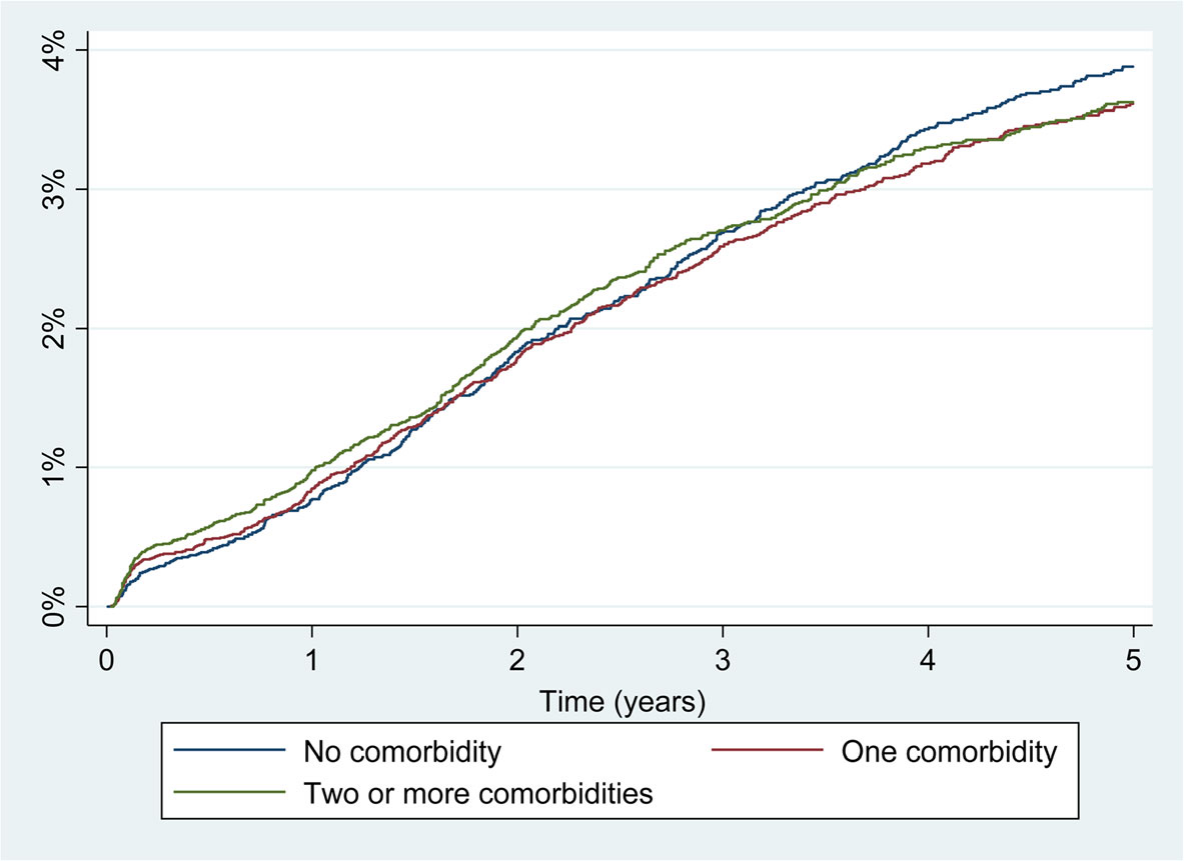
Incidence of revision by burden of comorbidity in TKA

Table 3 shows the results for TKA. Regarding the revision burden (Fig. 2), a higher incidence of revision was observed when the number of comorbidities was high, both at 1 and at 5 years’ follow-up, but the difference was only significant at 1 year and for 2 or more comorbidities, using 0 comorbidities as the reference category. With respect to specific comorbidities, after adjusting for patient characteristics, only obesity (SHR: 1.53; 95% CI: 1.17–1.99), deficiency anemia (SHR: 1.92; 95% CI: 1.18–3.11) and liver diseases (SHR: 2.50; 95% CI: 1.49–4.20) were related to an increase in revision risk at 1 year follow-up. The risk of revision at 5 years’ follow-up only remained significant for liver diseases (SHR: 1.60 95% CI: 1.11–2.29). Of the other comorbidities that compose the Elixhauser index, only the presence of paralysis was related with an increased revision risk at 5 years (SHR: 3.14; 95% CI: 1.49–6.60).

**Table 3:** Cumulative incidence of revision at 1 and 5 years’ follow-up and effect of comorbidities from the Elixhauser index on the risk of revision in Total Knee Arthroplasty (TKA)

	1 year follow-up					5 years’ follow-up				
	np	nr	incidence (95% CI)	SHR (95%CI)	***p value***	np	nr	incidence (95% CI)	SHR (95% CI)	***p value***
**Burden of comorbidity**					0.008					0.403
0 comorbidities	13,139	107	0.77 (0.63–0.92)	1.00		7532	333	3.74 (3.40–4.10)	1.00	
1 comorbidity	15,838	142	0.84 (0.71–0.99)	1.18 (0.93–1.53)		8533	355	3.48 (3.18–3.79)	1.04 (0.92–1.19)	
> =2 comorbidities	15,465	164	0.97 (0.83–1.13)	1.39 (1.09–1.78)		7505	321	3.47 (3.17–3.79)	1.06 (0.93–1.20)	
**Uncomplicated hypertension**	24,118	228	0.88 (0.77–1.00)	1.13 (0.93–1.37)	0.214	12,479	511	3.38 (3.14–3.63)	1.03 (0.93–1.15)	0.549
**Uncomplicated diabetes**	7005	60	0.79 (0.61–1.01)	0.92 (0.70–1.21)	0.541	3556	139	3.17 (2.74–3.62)	0.91 (0.78–1.06)	0.216
**Obesity**	4649	66	1.29 (1.00–1.63)	1.53 (1.17–1.99)	0.002	2329	94	3.78 (3.22–4.40)	0.97 (0.82–1.14)	0.711
**Depression**	4171	39	0.87 (0.63–1.17)	1.08 (0.77–1.51)	0.651	1929	101	3.93 (3.30–4.61)	1.06 (0.89–1.27)	0.484
**Chronic pulmonary disease**	3624	32	0.82 (0.57–1.14)	0.95 (0.66–1.36)	0.783	1795	76	3.25 (2.68–3.90)	1.01 (0.83–1.23)	0.933
**Hypothyroidism**	2524	24	0.85 (0.56–1.24)	1.12 (0.73–1.71)	0.603	1152	58	3.78 (3.02–4.67)	1.04 (0.83–1.30)	0.724
**Deficiency anemia**	990	17	1.58 (0.96–4.46)	1.92 (1.18–3.11)	0.008	457	15	3.34 (2.32–4.65)	1.09 (0.77–1.56)	0.615
**Renal failure**	775	9	1.02 (0.51–1.88)	1.25 (0.64–2.42)	0.515	262	9	2.40 (1.46–3.72)	0.98 (0.61–1.57)	0.938
**Hypertension with complications**	758	9	1.03 (0.511.89)	1.35 (0.70–2.61)	0.376	256	8	2.38 (1.41–3.75)	0.94 (0.58–1.53)	0.815
**Valvular disease**	745	10	1.19 (0.61–2.11)	1.61 (0.85–3.03)	0.144	365	9	2.66 (1.64–4.07)	0.94 (0.60–1.49)	0.803
**Rheumatoid arthritis**	572	8	1.27 (0.60–2.39)	1.37 (0.68–2.78)	0.379	235	10	3.65 (2.20–5.65)	0.90 (0.57–1.44)	0.673
**Other neurological disorders**	625	7	1.04 (0.47–2.06)	1.23 (0.58–2.59)	0.590	313	12	3.19 (1.98–4.85)	1.01 (0.64–1.60)	0.951
**Liver disease**	563	15	2.29 (1.34–3.66)	2.50 (1.49–4.20)	0.001	257	16	5.51 (3.81–7.66)	1.60 (1.11–2.29)	0.011
**Congestive heart failure**	432	3	0.66 (0.18–1.80)	0.88 (0.28–2.74)	0.882	226	5	2.00 (0.94–3.78)	0.70 (0.35–1.39)	0.306
**Peripheral vascular disorders**	338	4	1.11 (0.37–2.67)	1.35 (0.51–3.62)	0.545	198	7	3.62 (1.90–6.21)	1.16 (0.64–2.09)	0.626
**Solid tumor**	204	1	0.49 (0.05–2.50)	0.53 (0.08–3.79)	0.530	87	1	1.20 (0.23–3.97)	0.38 (0.10–1.57)	0.187
**Diabetes with complications**	191	2	0.97 (0.19–3.20)	1.08 (0.27–4.34)	0.911	78	8	5.71 (2.91–9.86)	1.82 (0.98–3.38)	0.057
**Coagulopathy**	190	2	0.93 (0.19–3.06)	1.10 (0.27–4.42)	0.896	85	1	1.46 (0.40–3.92)	0.53 (0.17–1.67)	0.280
**Blood loss anemia**	201	2	0.93 (0.19–3.07)	0.75 (0.19–3.12)	0.709	96	10	6.56 (3.56–10.80)	1.54 (0.87–2.77)	0.141
**Fluid and electrolyte disorders**	184	1	0.50 (0.05–2.58)	0.57 (0.08–4.12)	0.575	62	4	2.73 (1.03–5.89)	0.91 (0.37–2.18)	0.841
**Alcohol abuse**	171	2	1.07 (0.21–3.52)	0.85 (0.21–3.48)	0.827	76	4	3.79 (1.54–7.69)	0.91 (0.37–2.23)	0.841
**Psychosis**	155	0	NR	NC	NC	54	3	2.49 (0.67–6.58)	0.60 (0.19–1.86)	0.378
**Paralysis**	79	2	2.36 (0.45–7.42)	2.67 (0.76–10.78)	0.169	31	5	11.79 (4.98–21.78)	3.14 (1.49–6.60)	0.003
**Pulmonary circulation disorders**	58	1	1.41 (0.12–6.72)	1.87 (0.26–13.46)	0.533	27	1	3.09 (0.58–9.58)	1.05 (0.25–4.30)	0.951
**Lymphoma**	31	0	NR	NC	NC	12	1	3.49 (0.26–15.09)	1.16 (0.17–8.10)	0.881
**Peptic ulcer disease**	28	1	3.57 (0.26–15.41)	3.25 (0.45–23.51)	0.243	16	0	NR	NC	NC
**Drug abuse**	10	0	NR	NC	NC	3	0	NR	NC	NC
**Metastatic cancer**	9	0	NR	NC	NC	3	0	NR	NC	NC
**Weight loss**	8	0	NR	NC	NC	6	1	11.11 (0.6–38.77)	3.32 (0.46–23.81)	0.232
**AIDS**	4	0	NR	NC	NC	1	0	NR	NC	NC

*np* number of primary procedures at 1 and 5 years’ follow-up, *nr* number of revision procedures since the primary operation (from the beginning to 1 year and from 1 year to 5 years, at follow-up), *SHR* Subhazard ratio from competing risks models adjusted for sex, age, year of intervention, fixation type and type of hospital, *NR* no revisions undergone from 1 to 5 years’ follow-up, *NC* Not calculable

Incidence (95%CI): cumulative incidence of revision considering patient death as a competing event and the 95% Confidence Interval

Variance inflation tests < 4 for all models

### Influence of comorbidity on the risk of revision in THA

**Fig. 3:**
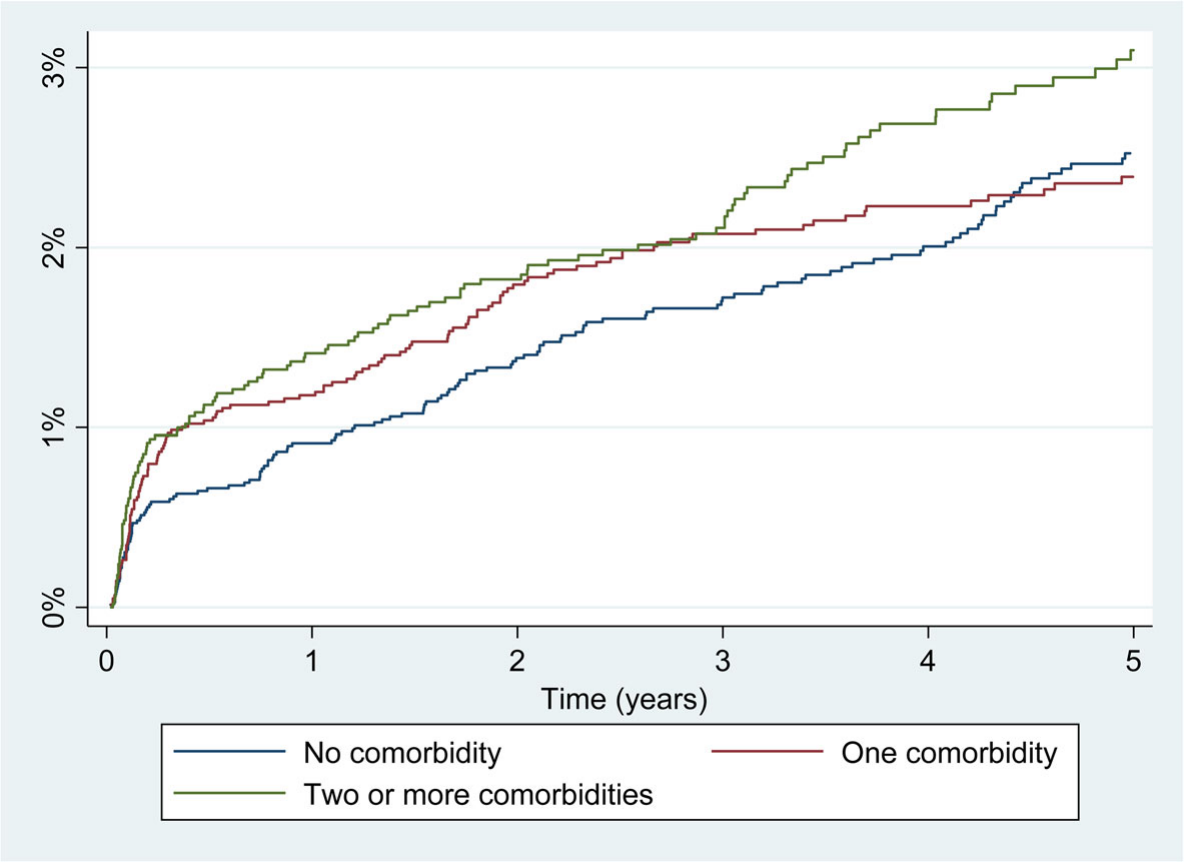
Incidence of revision by burden of comorbidity in THA

Looking at the incidence and risk of revision in THA (Table 4), the burden of comorbidity (Fig. 3), especially having 2 or more comorbidities, was shown to be significantly related to a higher risk of revision, both at 1 year (SHR: 1.51; 95% CI: 1.05–2.15) and at 5 years’ follow-up (SHR:1.32; 95% CI: 1.03–1.71). When focusing on the most frequent specific conditions in the Elixhauser index, only obesity was related to a higher revision risk, both at 1 year (SHR: 1.81; 95% CI: 1.17–2.80) and at 5 years’ follow-up (SHR: 1.80 95% CI: 1.31–2.47). Peripheral vascular disorders were also related to an increase in risk of revision at 5 years (SHR: 2.24; 95% CI: 1.06–4.74). The presence of metastatic cancer (only at 1 year) and the presence of psychosis (only at 5 years) might be related to an increase in the risk of revision. These results might be spurious due to the small sample sizes (*n* = 5 for metastatic cancer at 1 year and *n* = 17 for psychosis at 5 years).

**Table 4:** Cumulative incidence of revision at 1 and 5 years’ follow-up and effect of comorbidities from the Elixhauser index on the risk of revision in Total Hip Arthroplasty (THA)

	1 year follow-up					5 years’ follow-up				
	Np	Nr	Incidence (95% CI)	SHR (95%CI)	***p value***	Np	Nr	incidence (95% CI)	SHR (95% CI)	***P value***
**Burden of comorbidity**					0.021					0.030
0 comorbidities	6270	61	0.92 (0.71–1.18)	1.00		3391	79	2.45 (2.06–2.88)	1.00	
1 comorbidity	5501	70	1.17 (0.92–1.47)	1.29 (0.91–1.84)		2717	56	2.33 (1.95–2.77)	1.09 (0.85–1.40)	
> =2 comorbidities	4388	68	1.39 (1.10–1.76)	1.51 (1.05–2.15)		1927	53	2.94 (2.44–3.50)	1.32 (1.03–1.71)	
**Uncomplicated hypertension**	7161	100	1.29 (1.06–1.56)	1.21 (0.91–1.62)	0.189	3448	78	2.60 (2.23–3.00)	1.12 (0.91–1.38)	0.276
**Uncomplicated diabetes**	1949	31	1.43 (0.99–2.00)	1.30 (0.89–1.92)	0.177	901	24	2.91 (2.21–3.76)	1.25 (0.94–1.67)	0.130
**Obesity**	1072	23	1.90 (1.24–2.79)	1.81 (1.17–2.80)	0.007	479	21	4.39 (3.21–5.84)	1.80 (1.31–2.47)	< 0.001
**Depression**	1030	15	1.32 (0.77–2.12)	1.16 (0.69–1.97)	0.575	455	16	3.35 (2.30–4.72)	1.28 (0.89–1.85)	0.185
**Chronic pulmonary disease**	1270	20	1.42 (0.90–2.16)	1.32 (0.83–2.07)	0.240	564	11	2.52 (1.74–3.54)	1.07 (0.74–1.54)	0.729
**Hypothyroidism**	668	8	1.08 (0.51–2.05)	0.92 (0.45–1.90)	0.825	262	6	2.44 (1.36–4.05)	0.87 (0.51–1.50)	0.625
**Deficiency anemia**	421	6	1.28 (0.54–2.65)	1.14 (0.51–2.53)	0.752	187	6	2.91 (1.58–4.90)	1.26 (0.71–2.23)	0.421
**Renal failure**	323	4	1.04 (0.35–2.51)	0.85 (0.31–2.33)	0.753	100	2	1.84 (0.75–3.84)	0.84 (0.39–1.89)	0.667
**Hypertension with complications**	254	1	0.33 (0.03–1.72)	0.27 (0.04–1.94)	0.192	69	1	0.75 (1.50–2.51)	0.36 (0.09–1.45)	0.151
**Valvular disease**	259	2	0.69 (0.14–2.31)	0.55 (0.14–2.20)	0.399	113	0	NR	NC	NC
**Rheumatoid arthritis**	249	3	1.06 (0.30–2.87)	0.93 (0.30–2.90)	0.898	101	4	2.88 (1.27–5.62)	1.16 (0.55–2.45)	0.690
**Other neurological disorders**	212	3	1.29 (0.36–3.49)	1.17 (0.38–3.67)	0.780	88	2	2.23 (0.84–4.85)	1.07 (0.44–2.59)	0.882
**Liver disease**	245	2	0.71 (0.14–2.38)	0.71 (0.18–2.87)	0. 630	106	5	3.42 (1.68–6.69)	1.23 (0.59–2.59)	0.581
**Congestive heart failure**	131	2	1.36 (0.27–4.42)	1.10 (0.27–4.51)	0. 895	52	0	NR	NC	NC
**Peripheral vascular disorders**	143	3	1.85 (0.51–4.93)	1.73 (0.54–5.40)	0. 349	67	4	5.39 (2.31–10.38)	2.24 (1.06–4.74)	0.034
**Solid tumor**	87	1	1.08 (0.09–5.25)	0.87 (0.12–6.13)	0. 885	40	1	2.20 (0.42–6.97)	1.01 (0.25–4.06)	0.987
**Diabetes with complications**	55	0	NR	NC	NC	22	0	NR	NC	NC
**Coagulopathy**	71	0	NR	NC	NC	37	0	NR	NC	NC
**Blood loss anemia**	65	0	NR	NC	NC	39	0	NR	NC	NC
**Fluid and electrolyte disorders**	113	1	0.80 (0.07–3.99)	0.68 (0.10–4.81)	0.701	47	1	1.67 (0.32–5.36)	0.77 (0.19–3.10)	0.715
**Alcohol abuse**	110	2	1.69 (0.33–5.42)	1.81 (0.47–7.23)	0.404	48	0	NR	NC	NC
**Psychoses**	65	2	2.72 (0.52–8.49)	2.62 (0.65–10.63)	0.176	17	2	5.88 (1.89–13.23)	2.77 (1.03–7.49)	0.044
**Paralysis**	35	1	2.63 (0.20–11.79)	2.46 (0.36–16.87)	0.375	14	1	5.58 (1.00–16.41)	2.55 (0.63–10.28)	0.188
**Pulmonary circulation disorders**	17	0	NR	NC	NC	9	0	NR	NC	NC
**Lymphoma**	16	1	6.25 (0.41–24.69)	5.32 (0.75–37.33)	0.092	4	0	NR	NC	NC
**Peptic ulcer disease**	3	0	NR	NC	NC	3	0	NR	NC	NC
**Drug abuse**	23	0	NR	NC	NC	10	0	NR	NC	NC
**Metastatic cancer**	5	1	10.0 (0.57–35.81)	12.23 (1.46–102.46)	0.021	3	0	NR	NC	NC
**Weight loss**	6	0	NR	NC	NC	2	0	NR	NC	NC
**AIDS**	14	1	7,14 (0.45–27.52)	8.17 (0.82–56.81)	0.089	6	0	NR	NC	NC

*np* number of primary procedures at 1 and 5 years’ follow-up, *nr* number of revision procedures since the primary operation (from the beginning to 1 year and from 1 year to 5 years, at follow-up), *SHR* Subhazard ratio from competing risks models adjusted for sex, age, year of intervention, fixation type and type of hospital, *NR* no revisions undergone from 1 to 5 years’ follow-up, *NC* Not calculable

Incidence (95%CI): cumulative incidence of revision considering patient death as a competing event and the 95% Confidence Interval

Variance inflation tests < 4 for all models

## Discussion

Our study suggests that the effect of comorbidity burden on revision risk tends to be lower as time passes. In terms of the specific comorbidities that compose the Elixhauser index, the results obtained show that the influence of specific comorbidities on revision risk could depend on the joint operated on. Additionally, while patients with obesity have a higher risk of revision in the short-term, independent of the joint operated on, in the medium-term this risk is higher only in THA patients. These results could be helpful in interpreting the estimates of the incidence of revision in TKA and THA in the short-term, and they might also be relevant in enhancing clinical decision-making and patient consent.

As previous research has shown, comorbidity burden, both in TKA and THA, might be related to the incidence of revision in the short-term (1 year) but not in the medium-term (5 years) [13]. These results are congruent, at least partially, with ours and are likely related to revision for postoperative infection. Other studies have also illustrated that comorbidities, including obesity, could affect the presence of postoperative infection [25, 26], which usually occurs within the first year after arthroplasty and can culminate in the need for early revision. Therefore, future research using postoperative complications as an outcome and considering comorbidity as an explanatory factor could help clarify the influence of comorbidity on the risk of revision [25, 27, 28].

As previously pointed out in other reviews, both TKA and THA outcomes might be worse in obese patients than in non-obese [29–31] patients. These results are consistent with ours in THA but not in TKA. Nevertheless, similar to previous research, we found that the influence of obesity on the incidence of revision in patients undergoing TKA might be non-significant at the medium-term [32, 33]. Therefore, this evidence allows us to hypothesize that the influence of obesity on the incidence of revision might be different over time depending, on the joint operated on. While obesity could have a greater influence on the short-term incidence of revision, disappearing over time in TKA, this effect might be higher over time in THA. Furthermore, since obesity was taken from ICD codes, we were unable to stratify for it, e.g. using the BMI cut-off values. However, given that ICD codes are only identified if there is a specific medical diagnosis, we can ensure that obesity could be considered as a health problem on its own, in addition to being a risk factor for other diseases. Further research to test these hypotheses and taking other possibly related factors, like physical activity, diet, or others into account, might be useful in determining the specifics of the relationship between obesity and the risk of revision, particularly in TKA.

In terms of specific comorbidities, previous research has suggested an association between preoperative anemia and postoperative morbidity after TJA [34]. Additionally, concerning liver diseases, as some studies have shown, patients with these conditions could have a higher likelihood of hospital readmission and prosthesis revision, which corroborates our results [5, 35]. Still, it seems that these pathologies could present a challenge to the orthopedic field and steps should be taken to thoroughly inform patients of increased risks for postoperative complications if they suffer from liver diseases or preoperative anemia [36]. The results for paralysis are contradictory to the results from previous studies, which might be due to differences within the paralysis category itself [4, 37]. In the Elixhauser index, this category could include conditions other than cerebral palsy, which was the type of paralysis focused on. Therefore, to carry out further studies on patients with these conditions, it might be pertinent to have a better understanding of the relationship between paralysis and the risk of revision in TKA.

In terms of the impact of specific conditions in THA, peripheral arterial diseases were related to the risk of revision at 5 years. Though our results are statistically significant, other literature supporting this is lacking. Furthermore, as shown in previous research, a greater risk seems to be shown at 1 year revision than at 5 years [38], which contradicts the findings of this study. Concerning the lesser represented comorbidities, in THA there is a slight possibility of a relationship between increased risk of revision and metastatic cancer in the short-term, and psychoses in the medium-term, which has also been found in previous studies [13]. Regarding patients with metastatic cancer and psychosis, it should be noted that, due to the low number of patients in our study with these comorbidities, despite being significant, the relationships found could be spurious given the wide Confidence Intervals found. These results, focused on less common comorbidities, might serve as an exploratory analysis and starting point for further research. Studies with larger sample sizes could solve the issue of multiple testing with small sample sizes and might help improve the knowledge of the relationship of these less frequent pathologies and the risk of revision in THA. To shed light on this relationship, it might be important for patients and medical professionals to discuss and assess possible consequences when considering a THA in a patient that suffers from metastatic cancer and psychoses [20, 21, 39–41].

As limitations of this study, firstly, we should note that in some specific comorbidities the sample size was extremely small and stratifying the analyses by the reasons for revision was impossible. Despite this, it is important to mention that the results from conditions with small sample sizes, independent of the cause of revision, could serve as a starting point to establish hypotheses for further research including about the comorbidities related to the different causes of revision. We should also mention that some of the 95% CI found for less common conditions are too wide to extract sound conclusions regarding their relationship with the risk of revision. Despite this, these results might highlight potentially relevant associations to be tested when larger sample sizes are available. Another limitation that has to be taken into account is the completeness of the RACat. Though for primary procedures it could be considered high, about 90%, the percentage of revision procedures captured falls to about 70%. As the completeness in revision procedures is not as high as desired, this potentially yields biased results and an underestimation of the impact of comorbidity in revision rates. Furthermore, differences in completeness between hospitals and over time were also observed [23]. To combat the aforementioned limitations, the RACat became mandatory in 2017 and a retrospective search for information about unreported revision procedures and hospitals with lower completeness (identified through the MBD-HD) is continuously done within the RACat. As such, we expect an increase in the completeness of the data in the upcoming years, particularly for revision procedures and the identified hospitals with lower completeness rates. The retrospective search of unreported revisions, was based on ICD-9-CM codes for hip and knee revision procedures (00.70 to 00.87) but it should be done case by case because the MBD-HD does not contain information on laterality, which is essential to link the revision with its corresponding primary procedure, at least in bilateral arthroplasties.

It’s important to highlight that the levels of burden were based on tertiles according to the distribution of the Elixhauser index in our population, and not on the severity of the comorbidities included in the index. Therefore, we should mention that there were too few studies to take the severity or weighting of the Elixhauser index into account. Future studies to assign weights to the comorbidities included in the index as well as studies considering the severity of the diseases included may help improve the knowledge of how comorbidity influences the incidence of revision of knee and hip arthroplasties. Furthermore, specific combinations of comorbidities, like obesity (obtained from ICD codes and not from height and weight) and diabetes without complications, were not taken into account in this study. Beyond studying the burden of comorbidity and the specific conditions in the Elixhauser index, considering clusters of comorbidities could serve to better capture the clinical complexity of patients undergoing THA and TKA. However, it should be highlighted that our study assumes a previous step necessary to learn which comorbidities are related to the risk of revision, which could serve as a starting point for future research to investigate which comorbidities could be related with each other as well as with a combined risk of revision. Another limitation is the possible increase in comorbidities over time. While comorbidities are unlikely to change after 1 year, they may change over 5 years [42]. Taking these potential changes into account might be relevant in determining the effects of comorbidity on the risk of revision in THA and TKA. Lastly, it is important to point out that only the risk of revision was taken into account as an outcome in this investigation and that findings from a specific geographical location, like Catalonia, may not be transferable to other areas. Furthermore, potential confounders like social deprivation and some health-related habits were not included because they are currently not within the scope of the MBD-HD and RACat datasets. Future collaborative registry studies including these potential confounders and focusing on other outcomes like mortality and PROMs would provide healthcare professionals with more evidence for decision-making regarding best care options when faced with comorbid patients needing a THA or TKA procedure.

## Conclusion

In conclusion, our study shows that comorbidity burden has significant effects on the risk of short-term revision in both TKA and THA. These effects seem to be reduced over time after the primary arthroplasty procedure, especially in the knee. Regarding specific comorbidities from the Elixhauser index, obesity was found to have a significant effect on the risk of short-term revision in TKA and THA. In THA, this effect seems particularly relevant and increases over time. Furthermore, other specific comorbidities could be related to revision risk, depending on the joint. Therefore, including comorbidity burden and specific comorbidities as an adjustment when analyzing the incidence of revision might be helpful in improving the precision of the estimates in the short-term but not in the medium-term, particularly in TKA. Further research with larger sample sizes, longer follow-up periods and data from different arthroplasty registries should be done to confirm these results and explore the influence of the Elixhauser comorbidity index on other outcomes. This information could be relevant for clinical decision-making, patient-specific information and consent, and for improving the results of both TKA and THA procedures.

## Data Availability

Data used for this study is available under reasonable request by contacting Mireia Espallargues or Jorge Arias-de la Torre.
